# Chemometrics-driven discrimination of flue-cured tobacco aroma types *via* GC-MS/MS and multivariate analysis

**DOI:** 10.1039/d5ra02888d

**Published:** 2025-11-03

**Authors:** Hongjing Yang, Jiandong Zhang, Chen Liu, Kai Song, Yunzhen Gao, Jinbin Wei, Yanling Liu, Zhipeng Zang, Zhen Wang

**Affiliations:** a Technology Research and Development Center, Gansu Tobacco Industry Co. Ltd. Lanzhou 730050 China absorbance911@sina.com &361514540@qq.com

## Abstract

In this work, a novel classification model for flue-cured tobacco aroma types is presented by integrating chemometric modeling with quantitative aroma component analysis. Three representative types of flue-cured tobacco samples were selected for their distinct flavor profiles and commercial importance. Sensory characteristics were quantified by descriptive analysis of a trained panel. Gas chromatography-triple quadrupole tandem mass spectrometry (GC-MS/MS) was employed to rapidly identify the aroma components. The aroma types of flue-cured tobacco were studied using correlation analysis, hierarchical clustering, principal component analysis (PCA), and discriminant analysis. In total, 31 aroma components of flue-cured tobacco were identified by GC-MS/MS. Each flue-cured tobacco sample was first assigned an aroma style based on geographical origin and subsequently corroborated by the descriptive panel. Correlation analysis successfully identified compounds related to aroma substances and the descriptive analysis indices of flue-cured tobacco. Cluster analysis cleanly segregated the samples into the three predefined aroma types. Six principal components were extracted from the PCA to construct the discriminant model. Internal and cross-validation both confirmed the discriminant model's reliability and accuracy. This study evaluated the potential of using tobacco aroma components to distinguish and classify flue-cured tobacco aroma types.

## Abstract 2

In this work, a new chemometrics model to classify flue-cured tobacco aroma types based on 31 aroma components analyzed by GC-MS/MS. The model proved effective through multiple validation approaches.

## Graphical abstract

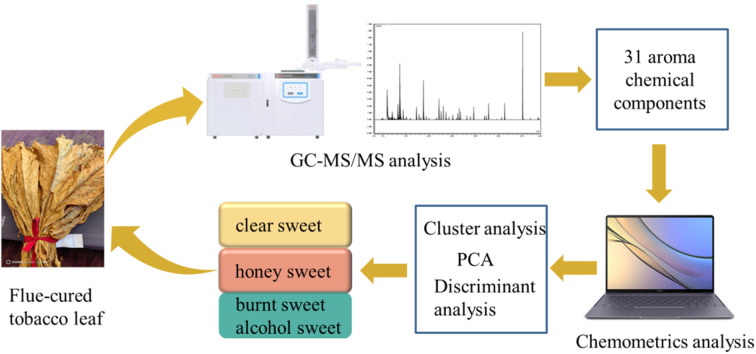

## 1 Introduction

Tobacco, a major global commercial crop, is cultivated in various countries, including China, the USA, and Brazil.^1^ Within the same tobacco variety, variations in geographical settings, climatic factors, and agricultural practices can lead to substantial disparities in both chemical and aromatic profiles, which in turn result in notable distinctions in the style and characteristics of the tobacco.^2,3^ In China, leveraging the ecological traits of the national flue-cured tobacco cultivation regions, Luo *et al.*^4^ identified eight distinct ecological zones. Correspondingly, they categorized the flavor profiles of tobacco leaves into eight specific types: clear sweet flavor, honey sweet flavor, alcohol-sweet flavor, burnt sweet burnt aroma, burnt sweet alcohol sweet flavor, clear sweet honey sweet, sweet burnt fragrance, and woody honey sweet flavor.^5^ For example, the tobacco leaves from Yunnan and Sichuan are considered to have a clear sweet flavor, the tobacco leaves from Guizhou are considered to have a honey sweet flavor, and the tobacco leaves from Hunan and Anhui are considered to have a burnt sweet alcohol sweet flavor.^6,7^ This classification highlights the complex interaction between geographical factors and the sensory attributes of tobacco.

The aroma type classification of flue-cured tobacco plays a critical role in guiding cigarette product development, processing techniques, and maintenance strategies. The classification of flue-cured tobacco leaves by geographical origin and sensory characteristics enables rapid identification of tobacco aroma types. However, this method also presents specific challenges and limitations that need to be addressed.^8^ Classification based on geographical origin typically focuses on the main tobacco-producing regions in China. In some less prominent tobacco-producing areas, the division is not apparent enough.^9,10^ Descriptive sensory analysis has long been the primary method for assessing the flavor of flue-cured tobacco. However, it faces limitations, including subjectivity, inter-evaluator variability, and an inability to provide detailed chemical insights into flavor profiles. Descriptive sensory analysis relies on human perception, which is susceptible to individual bias, environmental influences, and fatigue.^11–13^ Crucially, sensory methods fail to establish direct correlations between flavor attributes and specific chemical compounds, complicating flavor profile optimization and standardization.

Chemometric analysis, a discipline focused on the systematic collection, organization, analysis, and interpretation of data, is widely used in agricultural and food sciences, including principal component analysis (PCA), cluster analysis, and partial least squares discriminant analysis (PLS-DA).^14–16^ In the field of tobacco, Li *et al.*^5^ used partial least squares analysis (PLSA) to analyze the chemical composition of Shandong tobacco leaves, and identified 29 aroma precursors related to the sweet and burnt sweetness of Shandong tobacco leaves. Meng *et al.*^17^ applied correlation analysis and PLS to explore the relationship between tobacco leaf color characteristics and natural chromatography during baking. Jing *et al.*^18^ used GC-MS pseudotargeted metabolomics, combined with chemometrics, to examine the relationships between tobacco metabolites and tobacco leaf geographical location and yield. PCA results showed that the effect of geographical location on metabolites was greater than that of yield. Chemometric analysis can objectively classify tobacco grades, evaluate sensory quality, and identify varieties, achieving an accuracy of over 90%.^19–21^ Compared with professional classification, the statistical model-based classification of stoichiometric analysis takes less time, effectively reducing costs while ensuring work quality.^18,22^ Although chemometrics analysis has certain advantages, there remains a significant knowledge gap regarding how best to integrate these methods for flue-cured tobacco flavor classification.

In this study, a chemometrics model for classifying flue-cured tobacco aroma types was developed to effectively distinguish them based on quantitative analysis of aroma components. The tobacco leaves with clear sweet, honey sweet and burnt sweet alcohol sweet were selected as the research objects. The aroma components in tobacco leaves were rapidly and quantitatively analyzed using GC-MS/MS. By combining aroma components and chemometrics, a model was established to accurately identify three aroma types in flue-cured tobacco leaves. The established discriminant model for aroma types can classify flue-cured tobacco leaves based on the aroma components of unknown samples. The discriminant model provides essential data to identify flue-cured tobacco flavor types, thereby holding both practical and theoretical significance for this classification.

## 2 Experimental

### 2.1 Chemicals

All chemicals were used as received without further purification. *Trans*-2-hexenoic acid, N,O-bis(trimethylsilyl)trifluoroacetamide (BSTFA), dichloromethane (BCM), and acetonitrile (ACN) were procured from Ballantine Co., Ltd. Standards for aroma components were obtained from Aladdin Reagents Co., Ltd, with purities exceeding 95%. All reagents used in the study were of chromatographic grade.

### 2.2 Experimental materials

All tobacco varieties provided by the Gansu tobacco industry were the Yunyan 87 variety, harvested in 2022, as shown in Table S1. According to the relationship between origin and aroma type, these tobacco leaves were classified into aroma types.^23^ Samples A1 to C3 serve as the modeling dataset, whereas samples X1 to X3 constitute the validation dataset.

### 2.3 Descriptive analysis of flue-cured tobacco

Cigarette sample preparation: Tobacco leaf samples were moisture-adjusted, de-veined, shredded, and rolled into uniformly sized sticks using an automatic cigarette rolling machine (HAUNIBABY D-7300, BGWT, Germany). Prior to smoke sensory analysis, cigarette samples were equilibrated for 48 h at 25 ± 5 °C and 40 ± 5% relative humidity.^24^

The sensory analysis was conducted using a descriptive method, and the panel consisted of seven smoking assessors with at least 10 years of experience in cigarette sensory evaluation. The evaluation took place in a sensory room maintained at 18–25 °C and 55–70% relative humidity with good ventilation. The descriptive analysis indices of the cigarette samples were evaluated in accordance with the YC/T 530 standard.^25^ Descriptive analysis indexes included aroma quality, aroma quantity, diffusivity, satisfaction, saliva production sense, smoke concentration, offensive taste, irritancy, and aftertaste. Each descriptive analysis index was scored on a 5-point scale, with higher scores indicating better sensory quality. The concept of each descriptive analysis index is shown in Table S2.

### 2.4 GC-MS/MS analysis

The sample analysis was carried out using a TRACE1310–GC coupled to a TSQ8000E-MS/MS (GC-MS/MS, Thermo Fisher, USA). In the GC-MS/MS analysis, the liquid injection method was used, with an injection volume of 1 μL.

GC conditions were as follows: an Hp-5 MS chromatographic column (60 m × 0.25 mm × 0.25 μm) was used. The carrier gas was 99.999% pure helium, flowing at 1.5 mL min^−1^. The temperature gradient program was set as follows, with the column temperature initially maintained at 60 °C. It was then ramped to 95 °C at 5 °C min^−1^ and held for 2.5 min. Subsequently, the temperature was increased to 150 °C at a rate of 3 °C min^−1^, and held for 1 min. Finally, the temperature was ramped to 250 °C at 5 °C min^−1^. A split injection mode was employed with a split ratio of 3 : 1, and the split flow rate was set to 5 mL min^−1^.

The mass spectral conditions included an electron energy of 70 eV and an ion source temperature of 280 °C. The quadrupole temperature was 280 °C, and the transfer line temperature was 280 °C. The scanning mode was multiple reaction monitoring (MRM).

Qualitative and quantitative analysis was performed using ThermoFisher's Chameleon software. Compound identification was based on the NIST20 mass spectral library. Quantification by the internal standard method.^26^

### 2.5 Preparation of standard working solutions

The aroma standard was accurately weighed on an analytical balance (accuracy of ±0.0001 g), dissolved in DCM, and transferred to a 10 mL volumetric flask. Diluted to the mark with DCM to prepare a single standard stock solution of aroma substances. A 100 μL aliquot of each stock solution was combined in a new 10 mL volumetric flask. This mixture was diluted to the mark with DCM to prepare the mixed standard stock solution. Different volumes of the mixed stock solution and 50 μL of the internal standard (*trans*-2-hexenoic acid, 12.39 mg mL^−1^ in DCM) were added to 10 mL volumetric flasks. These solutions were diluted to volume with DCM/ACN (1 : 2, v/v) to generate a standard working solution. From each standard working solution, 1 mL was transferred into a chromatographic vial, followed by the addition of 100 μL of BSTFA. The mixture was reacted in a water bath at 60 °C for 40 min, cooled to room temperature, and subjected to GC-MS/MS analysis.^27^

### 2.6 Sample preparation

The sample pretreatment was carried out according to the literature report, and the corresponding modification was applied.^28,29^ Each tobacco sample (50 g) was crushed and sieved through a 0.25 mm aperture. One gram of tobacco powder was placed into a 50 mL centrifuge tube, and 1.5 mL of pH 3.0 PBS was added; then the mixture was allowed to stand for 20 min. Then, 50 μL of the internal standard solution and 10 mL of DCM were added. The mixture was vortexed at 2000 rpm for 20 min to facilitate extraction, then centrifuged at 6000 rpm for 10 min. The supernatant was filtered through a 0.45 μm organic phase filter membrane. Transfer the filtrate into a chromatographic vial to a volume of 1 mL, then add BSTFA to a volume of 100 μL. The vial was heated in a water bath at 60 °C for 40 min, cooled to room temperature, and then analyzed by GC-MS/MS.

### 2.7 Chemometrics analysis

All experimental samples were weighed at least three times, and each sample was prepared and analyzed independently to ensure the reliability of the results. Before statistical analysis, the data were standardized using the formula, 
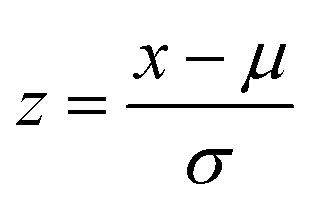
 (*x* = original value of the variable, *μ* = the variable's mean, *σ* = the variable's standard deviation).^30^ This standardization ensured all features operated on comparable scales. Statistical analyses were performed using Minitab17 software. In the cluster analysis, the association method was determined by selecting the sum of squared deviations. For measuring distances between clusters, Euclidean distance was chosen. In the principal component analysis, a correlation coefficient matrix of the matrix type was employed to assess the relationships among variables. In the context of discriminant analysis, the discriminant function used a linear model and cross-validation to assess predictive accuracy.

## 3 Results and discussion

### 3.1 Evaluation of the GC-MS/MS method

**Fig. 1:**
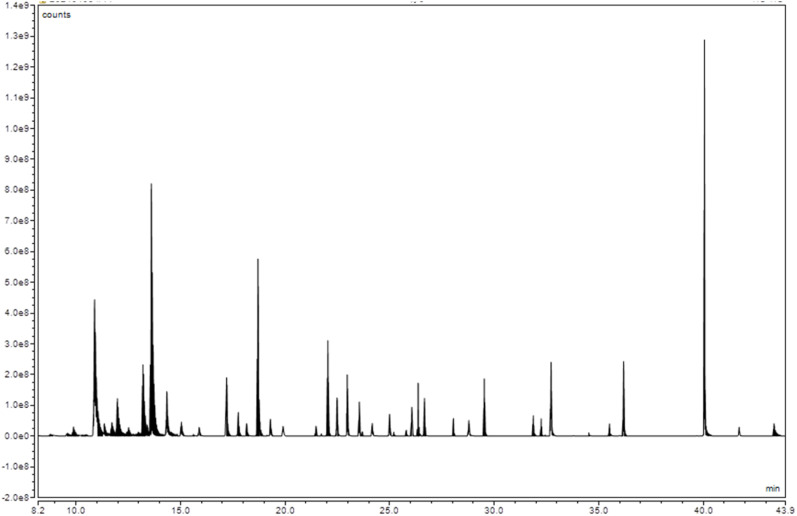
TIC of tobacco aroma components by GC-MS/MS analysis.

A novel GC-MS/MS method has been developed for determining the aroma components in tobacco. Fig. 1 presents the total ion chromatogram (TIC) profile obtained from the analysis. The retention times (RT) and corresponding qualitative/quantitative ion pairs for aroma components are detailed in Table S4. These ion pairs were carefully selected based on their relative abundance and specificity to ensure reliable identification and quantification of the target compounds. Method validation studies demonstrated satisfactory analytical performance (Table 1). All compounds exhibited excellent linear relationships within specific concentration ranges. The correlation coefficients (*R*^2^) for all compounds surpassed 0.99, indicating a high degree of linearity and reliability in the method's quantitative capabilities. The limit of detection (LOD) was determined based on a signal-to-noise (S/N) ratio of ≥3, while the limit of quantitation (LOQ) was established using an S/N ratio of ≥10.^31^ The low LOD and LOQ values obtained indicate that the method is highly sensitive and capable of detecting trace levels of aroma components in complex matrices (Table 1). The recovery (%) was calculated by the standard addition method. An analyte of known concentration was added to the tobacco sample, and then the recovery rate was calculated using the formula: [(sample spiked with standard-original sample)/spiked amount] ×100.^32^ The recovery of different flavor components was shown in Table 1, and the recoveries were in the range of 80 to 120%. These results collectively demonstrate that the developed GC-MS/MS method has high accuracy and precision, making it a powerful tool for analyzing aroma components in various samples.

**Table 1:** Evaluation indexes of 31 aroma components

Flavor composition	Standard curve/ng mL^−1^	*R* ^2^	Concentration range/ng mL^−1^	Recovery/%	LOD/ng mL^−1^	LOQ/ng mL^−1^
2-Methylbutyric acid	*y* = 0.0020*x* − 0.2281	0.9948	25–500	115.91	6.18	20.59
Pentanoic acid	*y* = 0.0068*x* − 0.1558	0.999	24–480	108.81	0.66	2.19
Hexyl alcohol	*y* = 0.0211*x* − 0.1109	0.9969	30–600	82.54	0.69	2.3
Dichromic acid	*y* = 0.0072*x* − 0.1485	0.9997	18.6–372	97.97	1.27	4.23
Lactic acid	*y* = 0.0031*x* + 0.6750	0.9996	125–2500	105.66	4.57	15.23
Hexanoic acid	*y* = 0.0014 − 0.0128	0.9974	100–1000	93.02	21.29	70.97
Hydroxyacetic acid	*y* = 0.0011*x* + 0.0503	0.9995	20.2–404	114.27	0.6	2.00
2-Methyl-2-pentenoic acid	*y* = 0.0007*x* + 0.0805	0.9986	90–1800	114.91	6.58	21.92
2-Methylcaproic acid	*y* = 0.0033*x* + 0.0383	0.9994	3–60	111.38	0.41	1.38
3-Hydroxypropionic acid	*y* = 0.0008*x* − 0.0366	0.9992	10–100	108.2	1.21	4.05
Benzyl alcohol	*y* = 0.0016*x* + 1.4841	0.9914	46.4–4640	85.41	2.26	7.54
2-Hydroxybutyrolactone	*y* = 0.0003*x* − 0.0078	0.9995	120–1200	89.58	1.67	5.58
Heptylic acid	*y* = 0.0061*x* − 0.0747	0.9993	20.4–408	118.39	1.97	6.58
Sorbic acid	*y* = 0.0020*x* − 0.0917	0.9995	25.8–516	108.31	7.51	25.05
2-Methylheptanoic acid	*y* = 0.0023*x* − 0.0917	0.9996	22.8–456	108.31	6.64	22.14
Guaiacol	*y* = 0.0142*x* + 0.0901	0.9996	25.2–504	90.93	0.59	1.97
Phenethyl alcohol	*y* = 0.0014*x* + 0.2255	0.9977	213–4260	113.58	2.28	7.61
*o*-Isopropylphenol	*y* = 0.0018*x* + 0.0345	0.9992	11.6–116	114.47	1.39	4.64
Benzoic acid	*y* = 0.0017*x* − 0.1046	0.9994	248–2480	118.43	45.67	152.22
3,4-Dimethylphenol	*y* = 0.0016*x* − 0.0678	0.9996	11.4–114	115.93	2.07	6.91
5-Hydroxymethyl-2(5*H*)furanone	*y* = 0.0021*x* − 0.0717	0.9924	50.5–505	111.89	0.32	1.05
4-Hydroxystyrene	*y* = 0.0012*x* − 0.1532	0.9952	105–1050	117.19	0.33	1.09
2,4, 6-Trimethylphenol	*y* = 0.0025*x* − 0.1149	0.9996	7.05–112.8	107.1	1.25	4.17
Carvacrol	*y* = 0.0094*x* − 0.2435	0.9984	14.8–185	91.48	1.37	4.58
*n*-Nonanoic acid	*y* = 0.0031*x* − 0.6836	0.9904	60–750	98.97	0.47	1.56
*n*-Decyl alcohol	*y* = 0.0071*x* − 0.0321	0.9972	10–100	87.96	0.03	0.1
*p*-Hydroxy benzaldehyde	*y* = 0.0009*x* − 0.0387	0.9948	52–832	88.72	1.2	4.00
Capric acid	*y* = 0.0055*x* − 0.7638	0.9904	18.4–368	91.34	0.27	0.89
4-Oxonaic acid	*y* = 0.0001*x* − 0.0029	0.9917	60.5–968	84.72	7.41	24.69
Cinnamic acid	*y* = 0.0010*x* − 0.0260	0.9956	116–1160	87.51	1.29	4.3
4-Hydroxyphenylethanol	*y* = 0.0112*x* − 0.8419	0.9959	516–6450	89.9	0.46	1.54

### 3.2 Aroma components in flue-cured tobacco

The extraction conditions for aroma components in tobacco and the derivatization reaction conditions were optimized, and the specific details were provided in the supplementary information. The determination of these aroma components was executed under the optimized conditions. Table S5 illustrates the contents of 31 aroma components identified in flue-cured tobacco samples from different types of flue-cured tobacco leaves.

These aroma components were classified by functional group, and the contents of the tobacco samples are presented in Table 2. Among them, sample X1 exhibited the highest content of aroma compounds, with a maximum value of 149.00 mg kg^−1^. Specifically, sample C2 had the highest content of acidic compounds, X3 contained the highest levels of phenolic compounds, and X1 had the highest content of alcohols. Moreover, X1 contained a notably greater diversity of four other types of compounds compared to the remaining 16 tobacco leaves.

**Table 2:** The content of aroma compounds in 17 flue-cured tobacco leaves (mg kg^−1^)

Sample ID	Phenols	Others	Alcohols	Acids	Sum
A1	4.6471	8.3683	51.6564	40.7771	105.4488
A2	3.6614	7.9645	37.0100	43.8389	92.4747
A3	4.8141	7.1333	46.4344	52.3358	110.7175
A4	2.7914	5.9239	28.7697	38.8871	76.3720
A5	4.5847	6.5969	44.4501	45.8834	101.5151
A6	3.4205	5.9454	29.4347	38.5491	77.3497
X1	7.8818	13.8674	62.4373	64.8193	149.0058
B1	2.8474	8.9802	26.7068	43.6927	82.2271
B2	4.3019	10.0445	11.5337	44.2407	70.1208
B3	3.3930	12.7480	13.7018	53.5056	83.3484
B4	3.5068	9.7178	33.9845	42.8406	90.0498
B5	4.1275	8.1158	42.9985	56.8090	112.0508
X2	4.7938	12.8534	34.7690	74.8703	127.2865
C1	5.0083	10.8484	44.1083	68.5724	128.5374
C2	4.4713	13.3675	35.3942	75.8308	129.0636
C3	4.3905	10.3242	35.1948	53.4571	103.3666
X3	12.4753	1.8237	31.3839	49.0707	94.7536

In the aroma classification of 17 flue-cured tobacco samples, classifications based on geographical origin and the descriptive analysis by a trained panel showed high consistency (Tables S1 and S3). The samples could be categorized into three aroma types. The samples A1 to A6 represented a clear, sweet flavor style; samples B1 to B5, a honey-sweet flavor style; and samples C1 to C3, a burnt-sweet, alcohol-sweet flavor style. Sample X1 was the clear sweet flavor style, sample X2 was the honey sweet flavor style, and sample X3 was the burnt sweet alcohol sweet flavor style. The aroma compounds of the three types of flue-cured tobacco are shown in Table 3. Among the three types of flue-cured tobacco leaves, burnt sweet alcohol sweet flavor exhibited the highest average content of both acidic and phenolic compounds. Meanwhile, the clear, sweet flavor had the highest average alcohol content. It was difficult to directly determine the relationship between the flue-cured tobacco aroma type and aroma content; multivariate analysis was performed to investigate potential patterns.

**Table 3:** Contents of chemical components in three aroma types of flue-cured tobacco leaves (mg kg^−1^)

Flavor composition	Clear sweet flavor	Honey sweet flavor	Burnt sweet alcohol sweet flavor
Acids	46.4415	52.6598	61.7328
Phenols	4.5430	3.8284	6.5863
Alcohols	42.8846	27.2824	36.5203
Others	7.9714	10.4099	9.0909
Sum	101.8405	94.1805	113.9303

### 3.3 Correlation analysis

To investigate the relationship between aroma components and aroma types in flue-cured tobacco, Pearson correlation analysis was employed. The correlation coefficient (*r*) and the corresponding significance level (*p* value) of each aroma type and volatile compound pair were calculated by binarizing the aroma type variables.^33^ When the |*r*| value is closer to 1, it indicates a stronger correlation. The results were shown in Table S6; the clear sweet aroma type showed strong negative correlations with 4-hydroxystyrene, 5-hydroxymethyl-2(5*H*)-furanone, heptylic acid, and *p*-hydroxybenzaldehyde. The honey-sweet aroma type exhibited strong correlations with 2-methylcaproic acid, 3, 4-dimethylphenol, phenethyl alcohol, and *n*-nonanoic acid. Burnt sweet alcohol sweet aroma type demonstrated strong positive associations with 4-hydroxystyrene, 5-hydroxymethyl-2(5*H*)-furanone, heptylic acid, and benzoic acid.

**Fig. 2:**
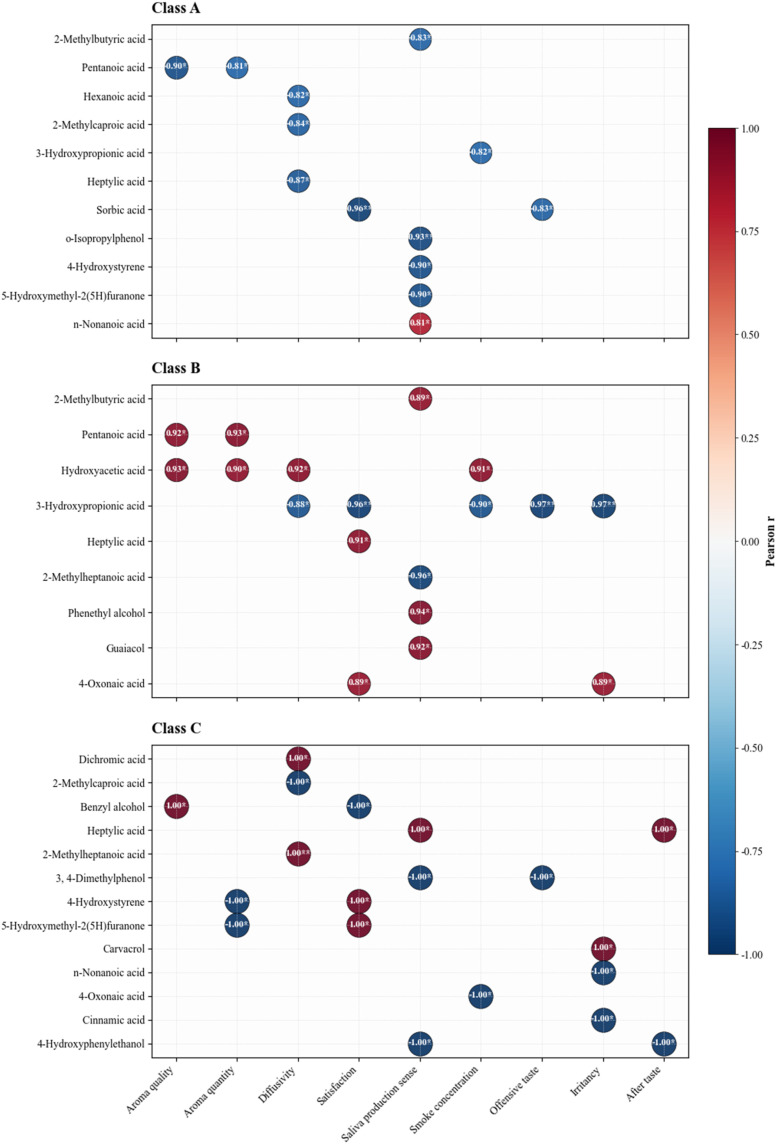
Pearson correlation analysis between sensory quality and aroma compounds in flue-cured tobacco. Clusters A, B, and C represent clear sweet flavor style, honey sweet flavor style, and burnt sweet alcohol sweet flavor style, respectively. Note: * indicates *p* < 0.05, ** indicates *p* < 0.01, and *** indicates *p* < 0.001, denoting statistically significant correlations.

The relationship between sensory attributes and aroma compounds in different aroma types of flue-cured tobacco was studied. Given that *p*-values are often hard to reach significant levels with small samples, this study focuses on statistically significant results (*p* < 0.05) to ensure the analysis is robust and credible. The results were presented in Fig. 2, and significant correlations emerged between multiple compounds and the specific sensory attributes. For instance, in clear sweet flavor tobacco, the aroma quality and quantity exhibited negative correlations with pentanoic and hydroxyacetic acid content. In honey-sweet flavor tobacco, smoke concentration intensity showed a positive correlation with hydroxyacetic acid content and a negative correlation with 3-hydroxypropionic acid content.

### 3.4 Cluster analysis

Cluster analysis, a statistical method, is used to categorize both samples and variables into distinct groups.^34,35^ The aim is to segment the data into distinct clusters, where samples within the same cluster are more similar and those in different clusters are more distinct. Cluster analysis was performed using the content of 31 aroma components in flue-cured tobacco as the dataset. The smaller the distance between classes, the greater the similarity of samples, and the greater the distance between classes, the better the clustering effect.^36^

**Fig. 3:**
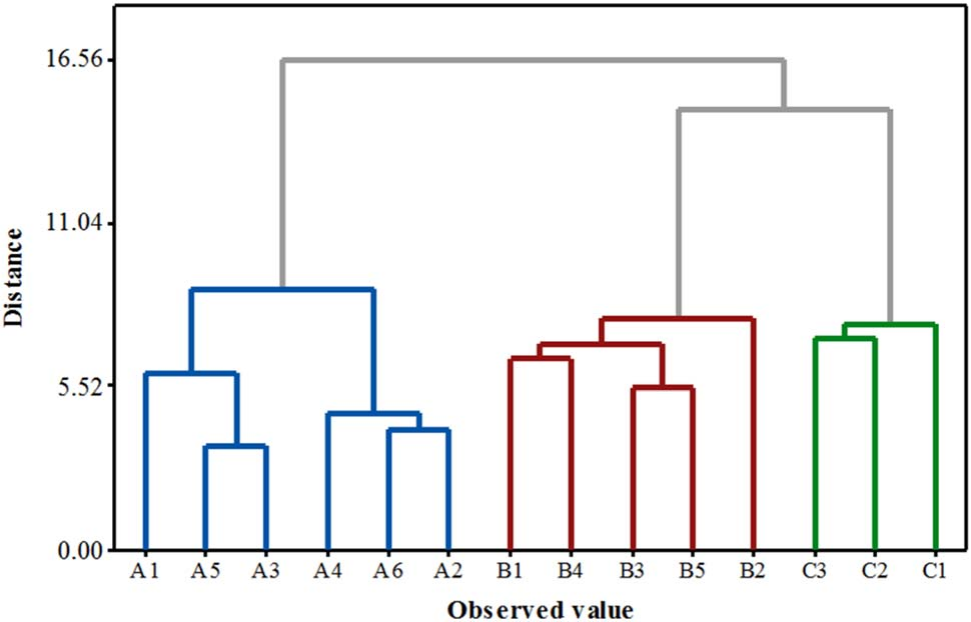
Cluster analysis diagram of flue-cured tobacco leaves.

The clustering results were shown in Fig. 3, with the horizontal axis representing the sample number and the vertical axis representing the distance metric. At a clustering distance of 11.04, the 14 tobacco samples were discernibly grouped into three distinct clusters. Sub-cluster 1 contained 6 samples with a clear sweet flavor, sub-cluster 2 included 5 samples of honey-sweet flavor tobacco leaves, while 3 samples from burnt, sweet alcohol sweet flavor were grouped into sub-cluster 3. These results of cluster analysis indicated that the aroma components of flue-cured tobacco were affected by aroma types, and different aroma types of flue-cured tobacco may be characterized by their aroma components.

### 3.5 Principal component analysis

PCA is a dimension reduction technique that transforms multiple variables into several factors, called principal components (PCs), which contain most of the information existing in the original data set.^37,38^ The PCA analysis of 31 aroma components in 14 samples was carried out to obtain the eigenvalue, contribution rate, and cumulative contribution rate of the correlation matrix. As shown in Table 4, the cumulative contribution rate of the first six PCs was 87.8%, indicating that these PCs accounted for 87.8% of the information in the original dataset. Therefore, the first six PCs were selected instead of the 31 original variables for subsequent discriminant analysis.

**Table 4:** Eigenvalue, variance contribution rate, and cumulative variance contribution rate of PCA

PC	Eigenvalue	Variance contribution rate%	Cumulative variance contribution rate%
PC1	10.615	34.2	34.2
PC2	7.376	23.8	58.0
PC3	2.913	9.4	67.4
PC4	2.426	7.8	75.3
PC5	2.297	7.4	82.7
PC6	1.577	5.1	87.8

**Fig. 4:**
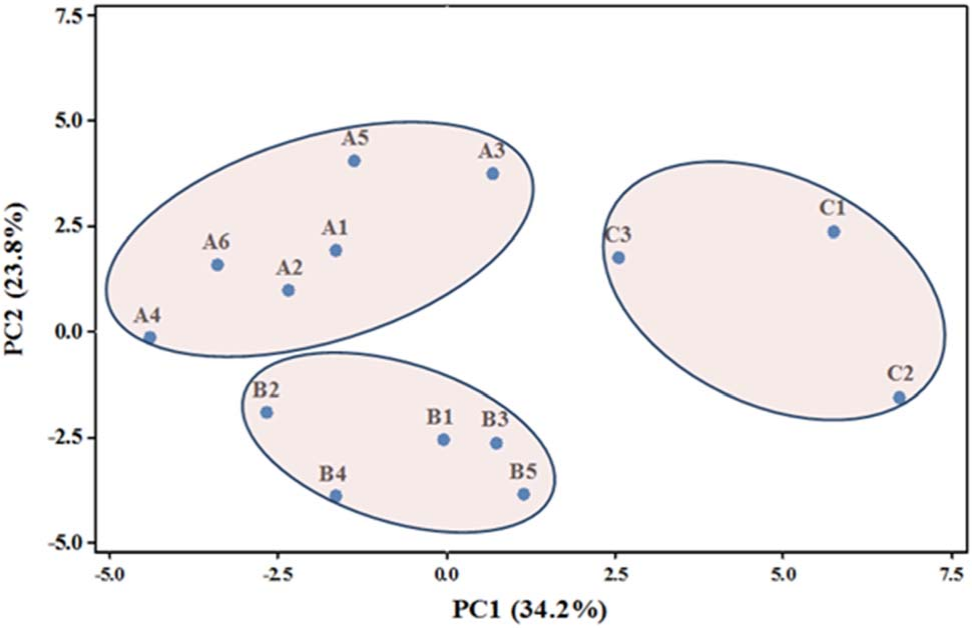
PCA projections of tobacco flavor components.

Both PC1 and PC2 were extracted, accounting for 34.2% and 23.8% of the variance, respectively (Fig. 4). The flue-cured tobacco samples from different flavor types and producing areas were clearly separated, indicating that their chemical components can be effectively distinguished.

The factor loadings of the principal components can be estimated from the correlations between the principal component scores and the original data. As shown in Table 5, the absolute values of the coefficients of variation of compounds heptanoic acid, 2-methyl-2-pentenoic acid, 4-hydroxystyrene, and 5-hydroxymethyl-2(5*H*)-furanone in PC1 were larger, indicating that they contributed more to this component. Similarly, 2-methylhexanoic acid, benzyl alcohol, phenethyl alcohol, and guaiacol showed higher absolute values of the coefficient of variation in PC2, indicating a greater impact on PC2.

**Table 5:** Principal component factor loading matrix

Flavor composition	*F*_1_	*F*_2_	*F*_3_	*F*_4_	*F*_5_	*F*_6_
2-Methylbutyric acid	0.4584	0.2287	−0.2638	0.6355	0.2416	−0.3485
Pentanoic acid	0.7300	0.4686	−0.2668	−0.0652	0.3355	−0.0111
Hexyl alcohol	0.6284	0.2192	−0.1307	0.2197	0.4653	0.2337
Dichromic acid	−0.2849	0.6009	0.4284	0.0046	0.2379	−0.2569
Lactic acid	0.4073	−0.5436	0.3921	0.2027	−0.2193	0.1679
Hydroxyacetic acid	0.6246	0.3907	0.3130	−0.3041	0.2799	0.3636
Hexanoic acid	0.7190	−0.1675	0.3077	−0.1127	−0.1561	0.4983
2-Methyl-2-pentenoic acid	0.9221	−0.0237	−0.0600	0.0548	−0.0268	0.2193
2-Methylcaproic acid	0.4611	0.7630	0.1435	−0.0523	−0.3205	0.1111
3-Hydroxypropionic acid	0.5646	−0.6056	0.3780	0.1208	−0.3243	0.1947
Benzyl alcohol	−0.1864	0.7578	−0.0674	0.3431	−0.1685	0.1793
2-Hydroxybutyrolactone	0.5440	−0.3817	0.2232	0.1617	−0.3809	−0.1222
Heptylic acid	0.8402	−0.0549	0.1889	−0.3412	0.2189	−0.0986
Sorbic acid	0.2085	−0.2974	−0.5019	0.1718	−0.4315	0.3020
2-Methylheptanoic acid	0.2340	0.3758	0.2529	−0.2554	−0.2490	−0.6200
Phenethyl alcohol	0.2790	0.9056	0.0014	0.2367	−0.0105	0.0964
*o*-Isopropylphenol	0.6134	−0.2986	0.4071	0.4285	−0.0065	−0.1550
Guaiacol	0.1925	0.9100	−0.0500	0.2781	0.0429	0.0816
Benzoic acid	0.8021	0.4672	−0.1362	0.0570	0.0804	−0.0954
3,4-Dimethylphenol	0.6111	0.7076	−0.0182	0.0970	−0.1745	0.0042
4-Hydroxystyrene	0.8820	−0.2316	0.2498	0.0505	−0.1546	−0.2231
5-Hydroxymethyl-2(5*H*)furanone	0.8828	−0.2281	0.2496	0.0517	−0.1560	−0.2219
2,4,6-Trimethylphenol	−0.2472	−0.5739	0.3295	0.2448	0.5898	0.0587
Carvacrol	−0.2382	−0.5318	0.3831	0.2325	0.6213	0.0702
*n*-Nonanoic acid	0.1240	−0.6879	−0.5739	−0.3019	0.0765	0.0346
*p*-Hydroxy benzaldehyde	0.7806	−0.4578	−0.2167	−0.1979	−0.0258	−0.0657
*n*-Decyl alcohol	0.6872	−0.3563	−0.4135	−0.1767	0.1352	−0.2848
Capric acid	0.7232	−0.0161	−0.3099	−0.4980	0.1532	−0.0774
4-Oxonaic acid	0.6909	0.2250	0.3195	−0.3576	0.3473	0.0778
Cinnamic acid	0.5698	−0.1315	−0.5639	0.3432	0.1823	0.1132
4-Hydroxyphenylethanol	0.4488	−0.5147	−0.1493	0.5867	0.0308	−0.1646

### 3.6 Establishment and verification of the discriminant model

Discriminant analysis is a statistical method that uses the known classifications of a sample set to derive a functional relationship that encapsulates them.^38,39^ To mitigate the impact of multicollinearity among independent variables on discrimination results, this study develops a discriminant analysis model based on PCA. Using the 14 modeling samples as the training set, the scores of the first six PCs were utilized as input variables. The resulting discriminant function was formulated as follows, and the prediction model for clear sweet flavor was presented below: *D*(*A*) = -7.453 − 3.181*F*_1_ + 2.894*F*_2_ − 1.591*F*_3_ + 0.909*F*_4_ − 2.188*F*_5_ + 2.929*F*_6_, the equation gave honey sweet flavor: *D*(*B*) = -3.827 + 0.868*F*_1_ − 2.442*F*_2_ + 0.234*F*_3_ − 0.105*F*_4_ + 1.563*F*_5_ − 1.190*F*_6_, burnt sweet alcohol sweet flavor was given by the equation: *D*(*C*) = −14.088 + 4.915*F*_1_ − 1.718*F*_2_ + 2.794*F*_3_ − 1.642*F*_4_ + 1.771*F*_5_ − 3.875*F*_6_. The samples were subjected to both self-verification and cross-validation to assess the model. The actual categories of the training samples, determined by descriptive analysis and origin classification, were entered into the model for analysis. As shown in Table 6, the self-verification method achieved 100% accuracy. All training set samples were correctly classified into their respective categories. Cross-validation achieved an accuracy of 85.7%, calculated based on the number of correctly classified samples (12 out of 14) divided by the total number of samples in the testing subset, as detailed in Table S7.

**Table 6:** The self-verification results of the training set

Observed value	Actual group	Prediction group	Group	Square distance	Probability
A1	A	A	A	6.326	1.000
			B	30.803	0.000
			C	57.678	0.000
B1	B	B	A	55.273	0.000
			B	7.536	1.000
			C	23.575	0.000
A4	A	A	A	5.818	1.000
			B	31.177	0.000
			C	89.343	0.000
B2	B	B	A	42.368	0.000
			B	5.491	1.000
			C	39.36	0.000
A6	A	A	A	1.563	1.000
			B	42.662	0.000
			C	86.127	0.000
A5	A	A	A	4.246	1.000
			B	61.289	0.000
			C	90.912	0.000
C3	C	C	A	62.316	0.000
			B	29.707	0.000
			C	3.446	1.000
A2	A	A	A	2.526	1.000
			B	48.43	0.000
			C	96.112	0.000
B3	B	B	A	25.268	0.000
			B	5.086	1.000
			C	42.616	0.000
C1	C	C	A	82.328	0.000
			B	34.583	0.000
			C	5.842	1.000
C2	C	C	A	99.657	0.000
			B	40.089	0.000
			C	5.952	1.000
A3	A	A	A	4.106	1.000
			B	40.455	0.000
			C	62.534	0.000
B5	B	B	A	53.204	0.000
			B	3.763	1.000
			C	38.229	0.000
B4	B	B	A	41.924	0.000
			B	4.300	1.000
			C	30.962	0.000

To rigorously evaluate the discriminant model's accuracy, three external validation samples were incorporated into the model. The PC scores were calculated using the corresponding matrix operations and employed as the sample group members for prediction purposes. The perfect consistency (100% cross-validation accuracy) between the external sample predictions (Table 7) and the expert panel evaluations attests to the model's predictive performance and its promising utility in practical settings.

**Table 7:** The results of the validation set

Observed value	Actual group	Prediction group	Group	Square distance	Probability
X1		A	A	14.059	0.631
			B	15.131	0.369
			C	53.287	0.000
X2		B	A	38.508	0.000
			B	19.016	0.976
			C	26.427	0.024
X3		C	A	46.996	0.000
			B	16.752	0.009
			C	7.302	0.991

To address the limitation of a small sample size, we replace the verification and training sets and adopt a strict external verification strategy. Group A samples were iteratively eliminated as external sets (A1–A6 excluded iteratively, supplement X1), group B samples as external sets (iteratively exclude B1–B5, increase X2), and group C samples (iteratively exclude C1–C3, supplement X3). For each iteration, the training set is used to re-PCA; these repeated PCA calculations are shown in Table S8. The first six PCs explained >85% of the cumulative variance, so they were retained to reconstruct the discriminant model. Subsequently, the model is applied to predict the excluded test sample categories. As shown in Table S9, while the model demonstrated strong overall accuracy (85.7%), performance varied across groups, with Group C showing notably lower prediction accuracy (33.3%). This limitation is primarily attributable to the small sample size in class C, which makes the model sensitive to class imbalance and reduces minority-class accuracy. While the limited sample size constrained the model's performance in Group C, this study establishes a foundational framework. It identifies key compounds relevant to aroma-type discrimination that merit further validation in larger-scale studies.

## 4 Conclusion

In this work, a discriminant model for flue-cured tobacco aroma types was developed based on aroma component analysis. Following optimization of extraction parameters and methodological validation, a GC-MS/MS-based approach was established to quantify 31 aroma compounds accurately. The aroma type of flue-cured tobacco samples was determined by dual verification of geographical origin and descriptive analysis using a trained panel. Correlation analysis identified aroma components associated with tobacco aroma profiles and sensory characteristics. Both cluster analysis and PCA effectively discriminated distinct flavor types of flue-cured tobacco leaves. Based on PCA results, a discriminant model for flue-cured tobacco aroma type was constructed. The discriminant analysis model demonstrated accurate performance across multiple validation methods. It should be noted that the sample size in this study, while sufficient for initial model construction, may limit the generalizability of the findings. Future work will prioritize expanding the dataset to include a broader range of geographical origins and harvest years to enhance the model's robustness and applicability.

## Author contributions

Hongjing Yang: methodology, resources, validation, formal analysis, discussion, writing -original draft, and writing–review and editing; Jiandong Zhang: methodology, supervision, writing and editing, and formal analysis; Chen Liu: methodology and formal analysis, validation, data curation; Kai Song: discussion and methodology, resources, validation; Yunzhen Gao: investigation, project administration, data curation; Jinbin Wei: software, investigation, resources; Yanling Liu: visualization, resources, validation; Zhipeng Zang: conceptualization, methodology, validation, supervision, discussion, and writing – review and editing; Zhen Wang: formal analysis, funding acquisition, resources, supervision, validation.

## Conflicts of interest

There are no conflicts to declare.

## Supplementary Material

RA-015-D5RA02888D-s001

## Data Availability

The data that support the findings of this study are available on request from the corresponding author. Supplementary information is available. See DOI: https://doi.org/10.1039/d5ra02888d.
