# Supplementary material for: Chemometrics-driven discrimination of flue-cured tobacco aroma types via GC-MS/MS and multivariate analysis
Source: RSC Adv. 2025 Nov 3;15(50):42354–63. doi: 10.1039/d5ra02888d (PMC12580854; doi:10.1039/d5ra02888d)
Supplement: RA-015-D5RA02888D-s001 [file RA-015-D5RA02888D-s001.pdf]

Supplementary Information (SI) for RSC Advances.
This journal is © The Royal Society of Chemistry 2025

Supplementary Information

Chemometrics-Driven Classification of Flue-Cured Tobacco Aroma Types via GC-MS/MS and Multivariate Analysis

The optimization of the experimental conditions

The aroma components in flue-cured tobacco samples were analyzed using a three-step method. Initially, the aroma components were extracted and separated from the tobacco leaf mixture. Subsequently, the extracted components underwent derivatization to enhance their chemical stability and analytical suitability. Finally, GC-MS/MS was employed to analyze the derivatized samples, enabling sensitive and accurate identification and quantification of the target aroma compounds. To optimize the method, several factors were carefully investigated and adjusted. These included the volume of buffer solution, the foaming time of the sample, the vortex time, the temperature and time of BSTFA derivatization.

In the experiment, the volume of buffer solution was optimized. As shown in **Figure S1 (A)**, the total content of the 31 aroma components in tobacco reached its highest level when the volume of the buffer solution was 1.5 mL. As the volume of the buffer solution increased beyond this volume, the content of the aroma components gradually decreased. Consequently, a buffer solution volume of 1.5 mL was chosen as the extraction solution for the aroma components in tobacco.

The impact of stand time on the extraction effect was examined within the range of 15 to 35 min. **As illustrated in Figure S1 (B)**, an initial rise was observed in the content of aroma components with increasing standing time, followed by a subsequent decline. The maximum total content of aroma components was achieved at a standing time of 20 min. Thus, 20 min was determined to be the optimal foaming time for maximizing the extraction of aroma components from tobacco.

The impact of vortex time on the extraction efficiency was evaluated. **As depicted in Figure S1 (C)**, within the range of 5 to 25 min, the content of aroma components showed a gradual increase with longer vortex time. Specifically, the highest content of aroma components was observed at 20 min of vortexing. However, considering the balance between efficiency and practicality, 20 min was determined to be the optimal vortex time for the extraction process.

As shown in **Figure S2 (A)**, it was evidenced that within the temperature range of 30-70 °C, the content of aroma components initially increased and then decreased with the rise in derivatization temperature. At 60 °C, the total amount of aroma components reached its peak. This

trend can be mainly attributed to the fact that low temperatures lead to incomplete derivatization, whereas high temperatures may cause some solvents to evaporate, thereby affecting the efficiency of the derivatization process. [1, 2] Therefore, 60 °C was selected as the optimal derivatization temperature of the experiment.

In addition, the impact of BSTFA derivatization time was examined, as illustrated in **Figure S2 (B)**. The content of aroma components peaked at 127.55 mg/kg when the derivatization time was 40 min. Beyond this point, extending the time did not significantly alter the content. Thus, 40 min was determined to be the optimal derivatization time.

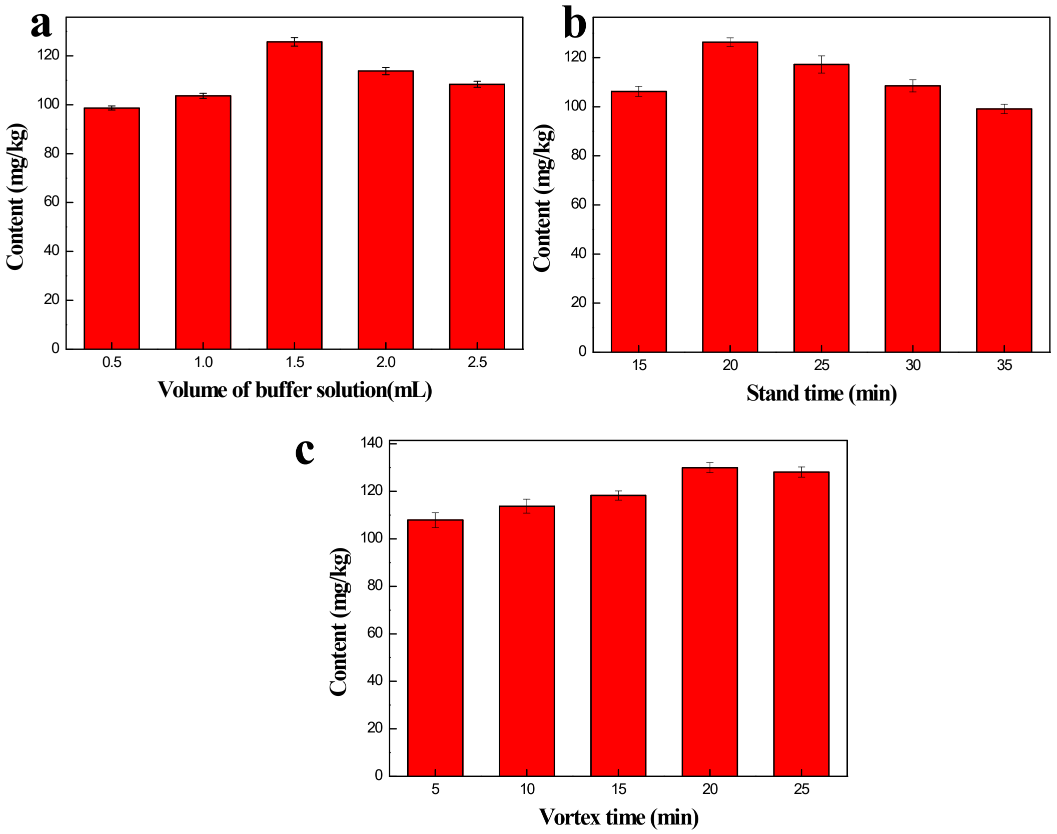

Figure S1 The influence of buffer volume (A), standing time (B) and vortex time (C) on the extraction effect

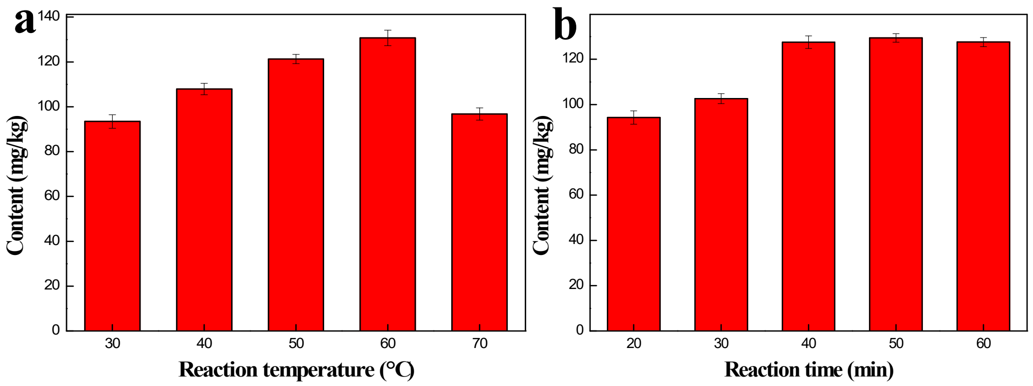

Figure S2 The influence of reaction (A) and reaction time (B) on the extraction effect

Table S1 Detailed information of the 17 flue-cured tobacco samples

Sample ID	Source/year	Style description
A1	Xuanwei C1F 2022	clear sweet flavor style
A2	Yuxi KHP 2022	clear sweet flavor style
A3	Huili C1F 2022	clear sweet flavor style
A4	Huidong C1F 2022	clear sweet flavor style
A5	Xuanwei B2F 2022	clear sweet flavor style
A6	Huili B2F 2022	clear sweet flavor style
B1	Yongzhou B2F 2022	honey sweet flavor style
B2	Chenzhou B2F 2022	honey sweet flavor style
B3	Yongzhou C1F 2022	honey sweet flavor style
B4	Ganzhou C1F 2022	honey sweet flavor style
B5	Chenzhou C1F 2022	honey sweet flavor style
C1	Zunyi C1F 2022	burnt sweet alcohol sweet flavor style
C2	Zunyi B2F 2022	burnt sweet alcohol sweet flavor style
C3	Anshun C1F 2022	burnt sweet alcohol sweet flavor style
X1	Yuxi C1F 2022	clear sweet flavor style
X2	Wuhu C1F 2022	honey sweet flavor style
X3	Tongren C1F 2022	burnt sweet alcohol sweet flavor style

Table S2 Descriptive analysis index and its concept description

Index	Concept
Aroma quality	The pros and cons of aroma and delicate degree, aroma quality good cigarette alcohol and, delicate, pleasant.[3]
Aroma quantity	The fullness of aroma and the degree of translucent, aroma quantity of cigarettes sufficient translucent, satisfying.[3]
Diffusivity	The degree of exposure and diffusion of aroma[4]
Satisfaction	The smoke is full-bodied, the aroma deep and layered, the strength perfectly balanced, and its persistence gratifyingly long—yielding complete satisfaction.[4]
Saliva production sense	The comfortable feeling of natural secretion of saliva in the mouth after the smoke exhaled from the mouth or nasal cavity.[5]
Smoke concentration	The initial feeling of the mouth when the smoke is just inhaled, which is not necessarily related to the aroma concentration.[6]
Offensive taste	Lack the essential smell of cigarettes, slight and obvious bad breath. Such as grass gas, raw gas, wood gas, local gas and so on.[7]
Irritancy	The slight and obvious discomfort caused by smoke to the senses. Such as the nasal cavity, mouth, throat sprint, etc.[7]
After taste	The residual taste perceived after smoke is exhaled from the mouth and nasal cavity, encompassing comfort, cleanliness, and dryness.[7]

Table S3 The sensory quality evaluation results for the flue-cured tobacco samples.

Sample ID	Style description	Aroma quality	Aroma quantity	Diffusivity	Satisfaction	Saliva production sense	Smoke concentration	Offensive taste	Irritancy	After taste
A1	clear sweet flavor style	4.04	4.04	4.00	4.03	3.70	3.07	3.54	3.68	4.03
A2	clear sweet flavor style	3.58	3.71	3.88	4.31	3.28	3.80	3.96	3.89	3.89
A3	clear sweet flavor style	3.17	3.33	3.75	4.17	3.75	3.75	3.89	3.89	4.17
A4	clear sweet flavor style	3.58	3.50	4.06	4.10	3.75	3.70	4.17	4.17	4.24
A5	clear sweet flavor style	3.50	3.63	3.50	4.38	3.75	3.85	3.89	4.10	4.10
A6	clear sweet flavor style	3.63	3.71	3.94	3.89	3.70	3.49	3.96	3.89	3.96
B1	honey sweet flavor style	4.25	4.21	4.38	3.75	4.38	2.97	4.24	4.24	4.44
B2	honey sweet flavor style	3.58	3.50	4.06	4.10	3.75	3.70	4.17	4.17	4.24
B3	honey sweet flavor style	3.58	3.71	3.88	4.31	3.28	3.80	3.96	3.89	3.89
B4	honey sweet flavor style	2.33	2.54	3.19	4.03	2.71	3.13	3.54	3.68	3.61
B5	honey sweet flavor style	3.53	3.57	3.88	4.24	3.23	3.70	3.96	3.89	3.89
C1	burnt sweet alcohol sweet flavor style	2.92	3.21	3.56	4.24	2.40	3.75	3.40	3.40	3.40

C2	burnt sweet alcohol sweet flavor style	2.79	3.04	3.44	4.51	2.81	3.70	3.61	3.68	3.54
C3	burnt sweet alcohol sweet flavor style	2.96	3.33	3.69	4.10	3.59	3.33	3.89	3.82	3.89
X1	clear sweet flavor style	3.08	3.33	3.75	4.17	3.33	3.39	3.89	3.96	3.89
X2	honey sweet flavor style	2.54	2.71	3.50	3.82	2.92	2.86	3.68	3.75	3.68
X3	burnt sweet alcohol sweet flavor style	2.81	3.15	3.62	4.42	2.69	3.70	3.50	3.50	3.61

Table S4 Qualitative and quantitative parameters for the determination of aroma components

Flavor composition	RT/min	Quantitative ion pair [m/z]	Impact energy [eV]	Qualitative ion pair [m/z]	Impact energy [eV]
2-Methylbutyric acid	10.06	75.0 / 47.0	10	143.0 / 75.0	10
Pentanoic acid	11.51	159.1 / 75.0	10	73.0 / 45.0	10
Hexyl alcohol	12.00	117.1 / 73.0	10	73.0 / 45.0	10
Dichromic acid	12.62	157.1 / 75.0	10	73.1 / 45.0	10
Lactic acid	14.38	117.1 / 73.0	10	73.1 / 45.0	10
Hydroxyacetic acid	15.07	75.0 / 47.0	10	117.0 / 75.0	10
Hexanoic acid	15.08	73.0 / 45.0	10	75.0 / 47.0	10
2-Methyl-*2*-pentenoic acid	15.62	73.0 / 45.0	10	73.0 / 45.0	10
2-Methylcaproic acid	15.93	73.0 / 45.0	10	187.1 / 75.0	10
3-Hydroxypropionic acid	18.1	155.1 / 81.0	15	155.1 / 53.0	10
Benzyl alcohol	18.65	135.0 / 107.0	15	165.0 / 91.1	10
2-Hydroxybutyrolactone	19.08	75.0 / 47.0	10	187.1 / 75.0	10
Heptylic acid	19.23	187.1 / 75.0	10	75.0 / 47.0	10
Sorbic acid	19.83	95.0 / 67.1	5	125.1 / 59.0	5
2-Methylheptanoic acid	20.00	95.1 / 67.0	5	125.1 / 59.0	5
Phenethyl alcohol	22.00	73.0 / 45.0	10	103.0 / 73.1	5
o-Isopropylphenol	22.39	73.0 / 45.0	10	193.1 / 73.1	10
Guaiacol	22.08	179.1 / 105.0	15	73.0 / 45.0	10
Benzoic acid	22.86	135.1 / 107.0	10	105.0 / 77.0	10
3, 4-Dimethylphenol	23.43	105.1 / 77.0	15	179.1 / 105.1	10
4-Hydroxystyrene	24.29	73.1 / 45.0	10	171.0 / 75.0	10
5-Hydroxymethyl-*2(5H)* Furanone	24.07	171.0 / 75.0	10	73.1 / 45.0	10
2,4, 6-Trimethylphenol	26.70	119.1 / 91.0	15	193.1 / 119.1	10
Carvacrol	26.65	208.1/193.1	15	193.1 / 119.1	10
n-Nonanoic acid	28.00	215.1 / 75.0	10	215.1 / 75.0	10

p-Hydroxy benzaldehyde	28.68	151.0 / 75.0	15	179.0 / 75.0	20
n-Decyl alcohol	28.75	151.1 / 95.1	10	179.0 / 151.1	10
Capric acid	32.2	117.0 / 75.0	10	229.1 / 75.1	15
4-Oxonaic acid	33.77	229.0 / 75.0	5	188.0 / 98.0	20
Cinnamic acid	35.51	205.1 / 131.1	10	161.1 / 145.1	5
4-Hydroxyphenylethanol	36.13	179.1 / 73.0	15	193.1 / 73.0	15
trans-*2*-hexenoic acid(**IS**)	15.40	171.00/ 97.00	5	171.00/129.00	5

Table S5 Content of aroma substances in tobacco leaves mg·kg^{-1}

Flavor composition	A1	A2	A3	A4	A5	A6	B1
2-Methylbutyric acid	4.8154	2.9118	3.7418	2.5682	2.9034	1.9744	2.1287
Pentanoic acid	2.4312	2.9877	4.7636	2.9114	3.3171	2.7980	3.1756
Hexyl alcohol	1.3705	0.6084	1.6729	0.9230	1.4988	0.9255	1.1677
Dichromic acid	1.4781	1.0434	1.0280	1.5349	1.3389	1.4173	0.8062
Lactic acid	10.6888	9.0143	8.4834	9.8460	10.9251	9.5868	9.2623
Hydroxyacetic acid	0.9233	0.7622	1.5201	1.0474	1.6270	0.9733	1.2385
Hexanoic acid	1.2904	1.3278	2.1870	1.5297	2.2611	1.6987	1.8819
2-Methyl-*2*-pentenoic acid	4.2027	8.8209	11.7300	4.1718	7.2763	3.7626	7.6244
2-Methylcaproic acid	0.3615	0.3561	0.3776	0.3165	0.3961	0.3678	0.2985
3-Hydroxypropionic acid	0.8563	0.8519	0.7823	0.7177	0.7267	0.7406	0.8445
Benzyl alcohol	1.2898	1.2949	2.7890	0.7389	4.8202	4.1064	0.4635
2-Hydroxybutyrolactone	2.3262	2.5798	1.7568	1.5705	1.6346	1.4593	2.0983
Heptylic acid	0.2435	0.2491	0.2749	0.2410	0.2769	0.2330	0.3195
Sorbic acid	0.4872	0.5151	0.4870	0.4803	0.4817	0.4937	0.4893
2-Methylheptanoic acid	0.4361	0.4250	0.4224	0.4169	0.4279	0.4362	0.4304
Phenethyl alcohol	7.3510	5.7678	8.8180	3.6448	8.6049	4.6857	2.6580
o-Isopropylphenol	1.1237	0.5467	0.8354	0.3374	0.7937	0.4376	0.6957
Guaiacol	1.7522	1.4118	2.0369	0.9178	2.0181	1.2789	0.7442
Benzoic acid	3.9444	4.6505	5.8114	3.4922	4.2089	4.0457	3.5591
3, 4-Dimethylphenol	0.8961	0.9341	1.1602	0.5400	1.0053	0.8626	0.4618

4-Hydroxystyrene	3.1820	2.7845	2.7412	2.1894	2.6352	2.3252	3.2806
5-Hydroxymethyl-*2(5H)*furanone	1.3435	1.1302	1.1068	0.8023	1.0460	0.8782	1.3920
2,4,6-Trimethylphenol	0.5869	0.5031	0.5134	0.6816	0.5013	0.5597	0.6482
Carvacrol	0.2881	0.2656	0.2683	0.3145	0.2664	0.2817	0.2974
n-Nonanoic acid	2.9079	3.6064	3.3519	3.4851	3.0870	3.4209	4.9450
p-Hydroxy benzaldehyde	1.5166	1.4701	1.5285	1.3617	1.2811	1.2826	2.2093
n-Decyl alcohol	0.1570	0.1516	0.1662	0.1485	0.1365	0.1402	0.2472
Capric acid	1.9709	2.0337	2.1823	2.0088	2.0177	2.2430	2.3360
4-Oxonaic acid	1.5715	1.6063	2.4240	1.6866	2.1629	1.5701	2.2281
Cinnamic acid	2.1677	2.6768	2.7682	2.4325	2.4487	2.7866	2.1244
4-Hydroxyphenylethanol	29.8804	21.1873	22.9883	23.3145	19.3897	19.5768	22.1705

Table S5 (Continued table)

Flavor composition	B2	B3	B4	B5	C1	C2	C3
2-Methylbutyric acid	3.9547	4.5187	2.4638	3.0477	6.5267	3.2679	2.6577
Pentanoic acid	2.9131	3.2147	1.6355	3.0687	6.1813	4.1564	3.9634
Hexyl alcohol	1.1791	1.5983	0.5654	1.8071	1.7202	1.4298	1.5931
Dichromic acid	1.4499	0.8903	0.8275	0.7234	1.2592	0.9425	1.6269
Lactic acid	10.0086	10.0202	15.2109	15.6528	10.2967	15.7643	11.5232
Hydroxyacetic acid	1.1128	0.9949	0.5276	1.2131	1.3337	1.8078	1.9187
Hexanoic acid	1.6271	1.8565	1.6109	3.0000	1.9589	5.9272	2.4855
2-Methyl-*2*-pentenoic acid	7.5779	12.8622	4.6917	10.3608	18.3176	23.9007	10.2600
2-Methylcaproic acid	0.2828	0.2927	0.3004	0.2965	0.3908	0.4113	0.3681
3-Hydroxypropionic acid	0.9003	0.9991	1.1049	1.0660	0.7847	1.3841	0.9800
Benzyl alcohol	2.0309	5.1078	0.3722	1.6954	3.2755	0.1614	4.5236
2-Hydroxybutyrolactone	2.3950	3.5610	2.8141	1.8314	2.3792	3.5589	3.0074
Heptylic acid	0.2772	0.2817	0.2621	0.2779	0.3288	0.3346	0.3480
Sorbic acid	0.4746	0.5451	0.4813	0.5028	0.4849	0.5065	0.4846
2-Methylheptanoic acid	0.4206	0.4222	0.4295	0.4196	0.4340	0.4262	0.4422

Phenethyl alcohol	3.4322	3.7650	2.4538	3.8485	7.6327	5.1644	6.5290
o-Isopropylphenol	0.9502	0.8742	1.2678	1.2036	1.0387	1.2304	1.1145
Guaiacol	1.0008	0.9677	0.6920	1.0621	1.8041	1.1247	1.4432
Benzoic acid	3.6674	4.3822	3.4277	4.1588	7.4428	5.7281	5.7128
3, 4-Dimethylphenol	0.4805	0.5344	0.5118	0.5573	1.4253	1.2399	0.9136
4-Hydroxystyrene	3.1379	3.5431	3.6583	3.0326	4.4152	5.1415	3.9397
5-Hydroxymethyl-*2(5H)* furanone	1.3164	1.5344	1.5954	1.2584	2.0082	2.4024	1.7500
2,4,6-Trimethylphenol	1.3749	0.7346	0.7122	0.9082	0.4782	0.5879	0.6211
Carvacrol	0.4955	0.2821	0.3229	0.3963	0.2619	0.2885	0.2982
n-Nonanoic acid	3.2321	4.5267	3.8341	4.5683	3.6099	3.4199	3.3111
p-Hydroxy benzaldehyde	1.3482	1.7702	1.6500	1.9935	2.0457	2.2648	1.6271
n-Decyl alcohol	0.1414	0.1831	0.1841	0.2152	0.2597	0.2078	0.1618
Capric acid	2.0107	2.1474	2.0045	2.1987	2.3744	2.3014	2.2337
4-Oxonaic acid	2.0255	1.7409	1.7940	1.9442	2.3279	2.4053	2.8070
Cinnamic acid	2.3054	3.8103	2.2343	4.3097	4.5202	3.1463	2.3341
4-Hydroxyphenylethanol	23.7240	29.1267	30.4091	35.4321	31.2202	28.4307	22.3872

Table S5 (Continued table)

Flavor composition	X1	X2	X3
2-Methylbutyric acid	4.4939	3.2361	3.1581
Pentanoic acid	2.5981	4.0674	3.1801
Hexyl alcohol	1.9038	1.3595	1.8899
Dichromic acid	1.6015	0.9482	0.7521
Lactic acid	19.4006	15.3108	14.6089
Hydroxyacetic acid	1.7128	1.7702	1.1247
Hexanoic acid	3.0831	6.0285	3.2350
2-Methyl-*2*-pentenoic acid	9.7634	23.5279	10.2698
2-Methylcaproic acid	0.5500	0.4111	0.3093
3-Hydroxypropionic acid	0.5276	1.3496	1.3800

Benzyl alcohol	1.3886	0.1364	0.1556
2-Hydroxybutyrolactone	3.4185	3.2176	0.2816
Heptylic acid	0.2557	0.3331	1.8549
Sorbic acid	0.2071	0.5203	1.9526
2-Methylheptanoic acid	0.2465	0.4171	0.2843
Phenethyl alcohol	7.5861	5.1431	0.4777
o-Isopropylphenol	1.9758	1.2108	4.1397
Guaiacol	1.6920	1.1051	5.7279
Benzoic acid	7.6437	5.9611	1.0685
3, 4-Dimethylphenol	2.1624	1.5409	1.2577
4-Hydroxystyrene	4.5718	5.0787	0.4830
5-Hydroxymethyl-*2 (5H)* Furanone	3.6906	2.3692	0.5949
2,4, 6-Trimethylphenol	1.6600	0.6361	1.0485
Carvacrol	0.3916	0.3009	0.3015
n-Nonanoic acid	3.5326	3.6182	1.0787
p-Hydroxy benzaldehyde	2.1865	2.1879	0.4642
n-Decyl alcohol	0.2265	0.1989	4.5320
Capric acid	0.2827	2.1548	0.7272
4-Oxonaic acid	2.4895	2.1457	2.1960
Cinnamic acid	6.4305	3.0702	1.8905
4-Hydroxyphenylethanol	35.3323	27.9311	24.3287

Table S6 Correlation between aroma type and aroma components of flue-cured tobacco

Flavor Composition	clear sweet aroma type	honey sweet aroma type	burnt sweet alcohol sweet aroma type
2-Methylcaproic acid	0.376	-0.871***	0.563*
4-Hydroxystyrene	-0.713**	0.042	0.811***
5-Hydroxymethyl-2(5H)furanone	-0.710**	0.039	0.811***
Heptylic acid	-0.702**	0.035	0.806***
3, 4-Dimethylphenol	0.219	-0.771**	0.636*
Benzoic acid	-0.176	-0.496	0.792***
Phenethyl alcohol	0.485	-0.744**	0.283
p-Hydroxy benzaldehyde	-0.694**	0.289	0.499
Guaiacol	0.527	-0.702**	0.184
2-Methyl-2-pentenoic acid	-0.474	-0.143	0.738**
n-Nonanoic acid	-0.530	0.716**	-0.196
2,4,6-Trimethylphenol	-0.442	0.675**	-0.255
4-Oxonaic acid	-0.432	-0.150	0.696**
n-Decyl alcohol	-0.644*	0.303	0.423
3-Hydroxypropionic acid	-0.637*	0.306	0.411
o-Isopropylphenol	-0.631*	0.282	0.432
2-Hydroxybutyrolactone	-0.607*	0.206	0.491
Pentanoic acid	-0.157	-0.416	0.676**
Capric acid	-0.462	-0.044	0.608*
Hydroxyacetic acid	-0.164	-0.385	0.648*
Carvacrol	-0.400	0.604*	-0.223

Hexanoic acid	-0.363	-0.128	0.587*
Dichromic acid	0.403	-0.578*	0.189
4-Hydroxyphenylethanol	-0.530	0.390	0.184
Lactic acid	-0.506	0.269	0.296
2-Methylheptanoic acid	-0.047	-0.351	0.467
Cinnamic acid	-0.356	0.092	0.321
Hexyl alcohol	-0.273	-0.050	0.389
2-Methylbutyric acid	-0.174	-0.106	0.333
Sorbic acid	-0.147	0.199	-0.055
Benzyl alcohol	0.088	-0.176	0.099

Note: * indicates $p < 0.05$, ** indicates $p < 0.01$, and *** indicates $p < 0.001$, denoting statistically significant correlations.

Table S7 The cross-validation results of the training set

Observed value	Actual group	Prediction group	Group	Square distance	Probability
			A	26.717	0.670
A1	A	A	B	28.127	0.330
			C	64.635	0.000
			A	157.574	0.000
B1**	B	C	B	74.492	0.000
			C	21.783	1.000
			A	20.848	0.980
A4	A	A	B	28.472	0.020
			C	105.228	0.000
			A	44.698	0.000
B2	B	B	B	20.745	1.000
			C	51.521	0.000
			A	2.466	1.000
A6	A	A	B	39.808	0.000
			C	82.138	0.000
			A	10.356	1.000
A5	A	A	B	89.8	0.000
			C	98.981	0.000
			A	63.21	0.000
C3	C	C	B	27.699	0.000
			C	13.3	1.000
			A	4.565	1.000
A2	A	A	B	49.447	0.000
			C	104.37	0.000
			A	26.905	0.010
B3	B	B	B	17.115	0.990
			C	58.541	0.000
C1**	C	B	A	96.119	0.000

			B	48.922	0.990
			C	58.748	0.010
			A	231.409	0.000
C2	C	C	B	80.309	0.000
			C	64.63	1.000
			A	9.735	1.000
A3	A	A	B	38.498	0.000
			C	61.086	0.000
			A	63.969	0.000
B5	B	B	B	9.338	1.000
			C	41.556	0.000
			A	41.226	0.000
B4	B	B	B	11.944	1.000
			C	29.702	0.000

Table S8 The cumulative variance contribution rate of PCA

Validation Scenario	Sample ID	PC1	PC2	PC3	PC4	PC5	PC6
Group A Exclusion	A1	0.3876	0.5661	0.7417	0.8118	0.862	0.9025
	A2	0.3777	0.5592	0.734	0.8032	0.852	0.8959
	A3	0.3931	0.5774	0.7305	0.7981	0.8516	0.8973
	A4	0.3713	0.557	0.7261	0.7954	0.8491	0.8956
	A5	0.392	0.5715	0.7256	0.7934	0.8471	0.8948
	A6	0.3697	0.5477	0.7213	0.7922	0.8467	0.894
Group B Exclusion	B1	0.3834	0.6066	0.7102	0.7897	0.8449	0.8923
	B2	0.3543	0.601	0.7011	0.7747	0.8392	0.893
	B3	0.3753	0.5958	0.6905	0.7757	0.847	0.9012
	B4	0.3813	0.5782	0.6771	0.7601	0.8354	0.8816
	B5	0.3785	0.5828	0.6836	0.7626	0.8386	0.893
Group C	C1	0.3621	0.5719	0.7351	0.8048	0.8596	0.909

Exclusion	C2	0.3942	0.6055	0.7522	0.8192	0.8699	0.9089
	C3	0.3637	0.5939	0.7471	0.8169	0.8693	0.9131

Table S9 The cross-validation results of external sets

External Test Set	Actual group	Prediction group	Group	Square distance	Probability
			A	1.285	1.0000
A1	A	A	B	47.4934	0.0000
			C	31.6224	0.0000
			A	0.6613	1.0000
A2	A	A	B	52.8908	0.0000
			C	38.7922	0.0000
			A	9.8237	0.9986
A3	A	A	B	56.3857	0.0000
			C	21.5201	0.0014
			A	19.1475	1.0000
A4	A	A	B	43.1888	0.0000
			C	51.6251	0.0000
			A	6.8637	1.0000
A5	A	A	B	96.5285	0.0000
			C	34.4306	0.0000
			A	1.7874	1.0000
A6	A	A	B	55.4861	0.0000
			C	49.7621	0.0000
			A	61.6298	0.0010

B1	B	B	B	0.9683	0.9990
			C	13.8562	0.0000
			A	25.4042	0.0001
B2	B	B	B	7.0385	0.9999
			C	30.2613	0.0000
			A	32.0592	0.0000
B3	B	B	B	8.3575	0.9999
			C	26.1163	0.0001
			A	58.2362	0.0000
B4	B	B	B	13.7307	1.0000
			C	36.0746	0.0000
			A	106.6067	0.0000
B5	B	B	B	5.0697	0.9987
			C	17.2845	0.0013
			A	2.3067	1.0000
C1*	C	A	B	29.2426	0.0000
			C	22.1876	0.0000
			A	145.9213	0.0000
C2	C	C	B	55.6898	0.3050
			C	53.0210	0.6950
			A	4.8395	0.9812
C3*	C	A	B	33.7701	0.0000
			C	11.3607	0.0188

Reference

[1]. O. Begou, K. Weber, B. Beckmann,D. Tsikas, *Molecules.* 2021, ***26***, 3206.

[2]. A. A. Ali, G. Bhat, I. Al-Ghamdi, W. Cao, A. Kumar, W. Iali, K. C. Narayan,Q. Ghazwani, *J Surfact Deterg.* 2024, ***27***, 605-612.

[3]. P. Huang, *J．Anhui Agric．Sci．*. 2015, ***43***.

[4]. C. N. T. M. Bureau, *YC/T 564-2018.* 2018.

[5]. B. E. Fowler, J. H. Ye, S. Humayun, H. Lee,L. J. Macpherson, *iScience.* 2022, ***25***.

[6]. J. Mai, Y. Ning, Z. L. Lin, H. Zhang, J. F. Sun, Y. Chen, Y.-L. Jiang, Y. F. Ma, M. J. Zhu,B. B. Hu, *Industrial Crops and Products.* 2025, ***234***, 121580.

[7]. SAC, *GB 5606.4-2005.* 2005.
